# Anti-Inflammatory Effects of *Huberia peruviana* Cogn. Methanol Extract by Inhibiting Src Activity in the NF-κB Pathway

**DOI:** 10.3390/plants10112335

**Published:** 2021-10-29

**Authors:** Seung A Kim, Chae Young Lee, Ankita Mitra, Haeyeop Kim, Byoung Young Woo, Yong Deog Hong, Jin Kyoung Noh, Dong-Keun Yi, Han Gyung Kim, Jae Youl Cho

**Affiliations:** 1 Department of Integrative Biotechnology, Sungkyunkwan University, Suwon 16419, Korea; seung-a26@naver.com (S.A.K.); chaeyoung2@skku.edu (C.Y.L.); rlagoduq7283@naver.com (H.K.); 2 Research Institute of Biomolecule Control and Biomedical Institute for Convergence at SKKU (BICS), Sungkyunkwan University, Suwon 16419, Korea; ankitamitra1710@gmail.com; 3 AMOREPACIFIC R&D Center, Yongin 17074, Korea; quddud@amorepacific.com (B.Y.W.); hydhong@amorepacific.com (Y.D.H.); 4 Instituto de BioEconomia, El Batan, Quito 170135, Ecuador; njk1201@hotmail.com; 5 International Biological Material Research Center, Korea Research Institute of Bioscience and Biotechnology, Daejeon 34141, Korea; lydian78@kribb.re.kr

**Keywords:** *Huberia peruviana* Cogn., methanol extract, NF-κB pathway, anti-inflammation, Src, acute gastritis, acute lung injury

## Abstract

There is a growing need to develop anti-inflammatory drugs to regulate inflammatory responses. An extract of *Huberia peruviana* Cogn. had the best inhibitory effect on nitric oxide (NO) production in screening process undertaken in our laboratory. However, the anti-inflammatory effect *of Huberia peruviana* Cogn. methanol extract (Hp-ME) has not been studied. In this study, the anti-inflammatory effect of Hp-ME was assessed by using an NO assay, RT-PCR, luciferase reporter gene activity assay, western blotting assay, HCl/EtOH-induced acute gastritis model, and LPS-induced acute lung injury model. The phytochemical components of Hp-ME were determined through LC-MS/MS analysis. When RAW264.7 and HEK293T cells were treated with Hp-ME, NO production was decreased dose-dependently without cytotoxicity and the mRNA levels of iNOS, COX-2, and TNF-α were decreased. In a luciferase assay, the activity of transcription factors, NF-κB in TRIF or MyD88-overexpressing HEK293T cells was extremely reduced by Hp-ME. The western blotting analysis indicated that Hp-ME has anti-inflammatory effects by inhibiting the phosphorylation of Src. Hp-ME showed anti-inflammatory effects on in vivo models of HCl/EtOH-induced gastritis and LPS-induced acute lung injury. LC-MS/MS revealed that Hp-ME contains several anti-inflammatory flavonoids. The final findings of this study imply that Hp-ME could be used as an anti-inflammatory drug in several inflammatory diseases.

## 1. Introduction

The inflammatory response in humans is a part of the innate immune system. When body tissue senses exogenous and endogenous danger signals such as bacteria, fungi, viruses, etc., inflammation is initiated [1]. It is a system for creating environmental conditions to fight challenges by activating the migration of leukocytes (neutrophils, monocytes, and eosinophils) and recruiting inflammatory mediators, including cytokines and chemokines [2]. Therefore, inflammation can maintain tissue homeostasis and play a major role in preventing infections [3].

Innate immunity initiates the activation of macrophages and dendritic cells to manage an immune response against pathogens and to act as an effective line of defense in humans, especially in the early stage of infection [4,5]. They are activated by stimuli such as pathogen-associated molecular patterns (PAMPs) [6]. The PAMP are common molecules possessed by the pathogens and recognized by pattern recognition receptors (PRRs) such as Toll-like receptors (TLRs) in immune cells [7]. Examples of pathogen recognition include the interaction between lipopolysaccharides (LPS) and TLR4, between Pam3csk4 and TLR1/2, and between Poly(I:C) and TLR3 [8]. When the TLR in the immune cell recognizes PAMPs, inflammatory signaling begins. The first step in the inflammatory signaling processes through TLRs is the activation of adaptor molecules called TIR-domain-containing adapter-inducing interferon-β (TRIF) and Myeloid differentiation primary response 88 (MyD88) [9]. Adaptor molecules can deliver signals by sequentially activating the kinase factors proto-oncogene tyrosine-protein kinase Src, IκB kinase α/β (IKKα/β), nuclear factor of kappa light polypeptide gene enhancer in B-cells inhibitor, alpha (IκBα), extracellular-signal-regulated kinase (ERK), and c-Jun N-terminal kinase (JNK). When these signals are finally delivered to the transcription factors, activator protein-1 (AP-1) and nuclear factor-κB (NF-κB) are present in the nucleus, and inflammation-related cytokines and enzymes are expressed [10]. As a result, mRNA expression levels of inducible nitric oxide (NO) synthase (iNOS), cyclooxygenase-2 (COX-2), and tumor necrosis factor-α (TNF-α) are increased. iNOS is an enzyme that catalyzes the reaction to produce NO from L-arginine; COX-2 is involved in the synthesis of prostaglandin; and TNF-α is an acute inflammation-related cytokine [11]. However, if inflammation responses continue to occur in the cell without stopping normally, problems that arise in organs can lead to disease [12]. Therefore, a study for the control of excessive inflammatory reactions would be useful.

An extract of *Huberia peruviana* Cogn. was among the many extracts belonging to an extract library with an anti-inflammatory effect in our laboratory. Specifically, it was the extract with the best inhibitory effect in the screening process for the inhibition of NO, the final product of inflammation. *Huberia peruviana* Cogn. is mainly collected in Ecuador and is especially found in Loja province [13]. While it has the potential to modulate excessive inflammatory responses, understanding levels of its anti-inflammatory effect and mechanism are rare yet. Therefore, we have studied the anti-inflammatory effects of *Huberia peruviana* Cogn. methanol extract (Hp-ME) using LPS/TLR4-activated macrophages and murine in vivo models of HCl/EtOH-induced gastritis and LPS-induced acute lung injury (ALI).

## 2. Results

### 2.1. Hp-ME Reduces NO Production in Inflammatory Responses Mediated by TLR

**Figure 1:**
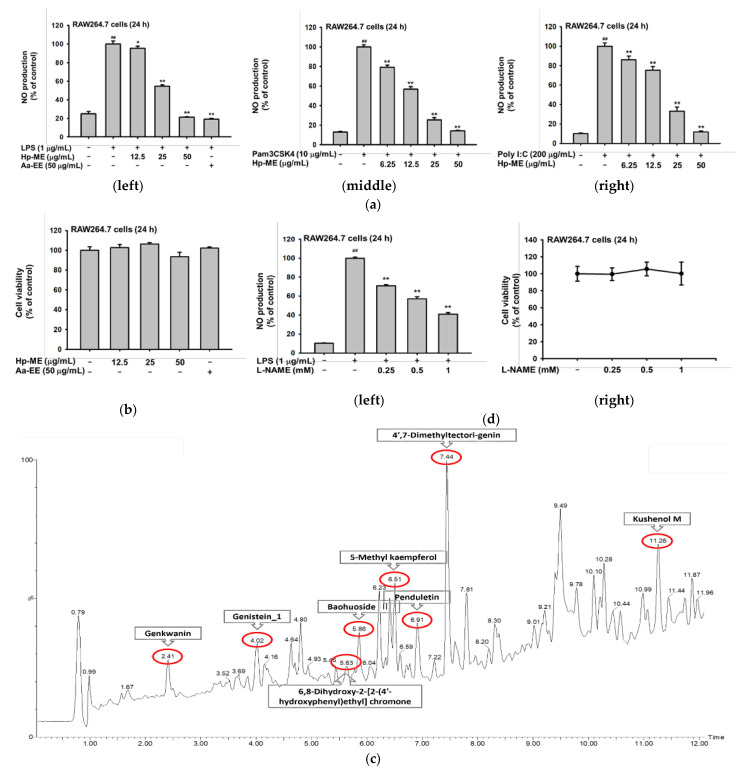
NO reductive effect of Hp-ME for murine macrophage. (**a**) NO production level when RAW264.7 cells were activated by LPS (1 μg/mL) (**left**), Pam3CSK4 (10 μg/mL) (**middle**), or Poly(I:C) (200 μg/mL) (**right**), without or with different concentrations of Hp-ME. The reductive effect of Hp-ME was compared to that of Aa-EE (50 μg/mL). (**b**) Viability in RAW264.7 cells was measured at several Hp-ME concentrations. (**c**) LC-MS/MS chromatogram of Hp-ME was analyzed. (**d**) The ability of the antagonist to produce NO (**left**) after L-NAME and cell cytotoxicity (**right**) were examined under conditions used in (**a**,**b**). All data (**a**,**b**,**d**) are presented as means ± standard deviation (SD) calculated from at least four independent samples. ##: *p* < 0.01 compared to the stimuli-untreated group, *: *p* < 0.05 and **: *p* < 0.01 compared to the control groups (treated with either LPS, Poly(I:C), or Pam3CSK4).

To confirm that Hp-ME has an anti-inflammatory effect, an NO assay was conducted to see whether it can inhibit the production of NO, the final product of inflammation. In the case of induction with LPS, the degree of NO generation decreased to 21% at 50 μg/mL compared to the 100% positive group (Figure 1a, left). Additionally, in Pam3CSK4 induction, it decreased by 14% (Figure 1a, middle), and with Poly(I:C), NO generation decreased by almost 11% (Figure 1a, right). When compared with Artemisia asiatica 95% ethanol extract (Aa-EE), the ability of Hp-ME to reduce NO is analogous to that of Aa-EE (Figure 1a, left). Next, the cell viability was not decreased below 90% (even when RAW264.7 cells were treated with Hp-ME for 24 h (Figure 1b)). Therefore, Hp-ME was not found to have cytotoxicity. Additionally, LC-MS/MS analysis was performed on constituents that allow Hp-ME to have an NO inhibitory effect. Several anti-inflammatory flavonoids have been found in Hp-ME. 5-methyl kaempferol, genistein 1, and penduletin are one of them (Figure 1c). In addition, to identify a flavonoid component, the NO inhibitory ability of Hp-ME was compared with NO production reduced by the NO inhibitor, L-NAME. In the same environment and with the same RAW264.7 cell line, the NO inhibition of Hp-ME increased in the same pattern as L-NAME in a concentration-dependent manner (Figure 1d, left). L-NAME also had no cytotoxicity and had similar viability as the control group (Figure 1d, right).

### 2.2. Hp-ME Has an Anti-Inflammatory Effect at the mRNA Expression and Transcriptional Level

**Figure 2:**
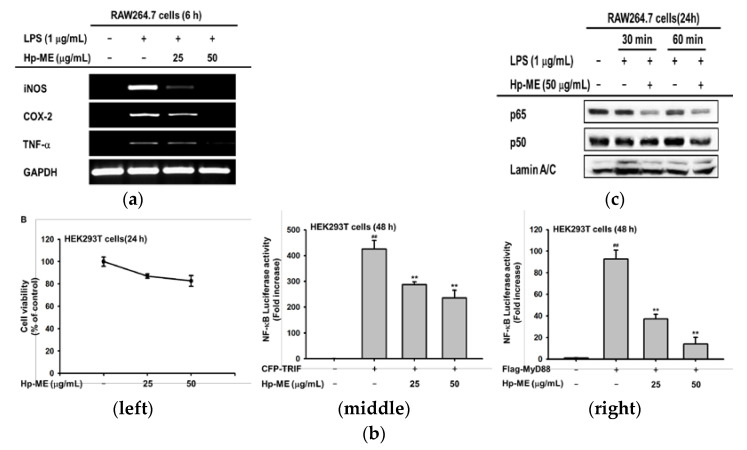
Inflammation inhibitory efficacy of Hp-ME at the mRNA expression level as well as at the transcriptional level for RAW264.7 and HEK293T cells. (**a**) The band intensity indicated mRNA expression level after LPS induction (6 h) in Hp-ME treated macrophages. The measured factors were iNOS, COX-2, and TNF-α. (**b**) Cell viability for HEK293T cells was also measured 24 h after Hp-ME treatment (**left**). The luciferase reporter gene activity was determined in co-transfected HEK293T cells by NF-κB luciferase reporter, β-galactosidase, CFP-TRIF (**middle**), and MyD88 (**right**) plasmids. After transfection (24 h), cells were treated with Hp-ME (25 and 50 μg/mL) for 24 h. (**c**) The protein expression levels of p65 and p50, subunits of NF-κB in the nucleus, were confirmed in nuclear lysates treated with Hp-ME (50 μg/mL) and LPS (1 μg/mL) for 30 and 60 min. Lamin A/C was a control protein. All data (**b**) are presented as means ± standard deviation (SD) calculated from at least four independent samples. ##: *p* < 0.01 compared to the un-transfected group, and **: *p* < 0.01 compared to the control group (only TRIF or MyD88 transfected group).

After confirming that Hp-ME suppressed NO production, we also checked iNOS mRNA expression level. The mRNA expression levels of COX-2 and TNF-α, which are enzymes and cytokines related to the inflammatory reaction, as well as iNOS, were decreased compared to the positive group treated with LPS. In addition, the bands totally disappeared at 50 μg/mL (Figure 2a). Next, to identify the anti-inflammatory effect of Hp-ME at the transcriptional level, assays were conducted to determine the luciferase activities of the transcription factor. First, cytotoxicity was checked in HEK293T cells and it was determined that Hp-ME was not toxic (Figure 2b, left). Furthermore, NF-κB Luciferase activity decreased in the Hp-ME treated group when TRIF, one of the adaptor molecules, was overexpressed. Transcription decreased to 60% at 50 μg/mL compared to the positive group (Figure 2b, middle). In this case, the NF-κB Luciferase activity declined to around 38% at 25 μg/mL and 17% at 50 μg/mL (Figure 2b, right). After affirming that the activity of the transcription factor was decreased by Hp-ME, the total protein levels of the two subunits of NF-κB (p65 and p50) were confirmed in the nuclear lysate. At 30 and 60 min after Hp-ME (50 μg/mL), the protein expression of p50 and p65 decreased. The degree was more noticeable with p65 (Figure 2c). These findings indicate that Hp-ME has an anti-inflammatory effect on mRNA expression as well as the activity of transcription factors.

### 2.3. Hp-ME Disrupts Phosphorylation of Src Kinase in the Intracellular NF-κB Signaling Pathway

**Figure 3:**
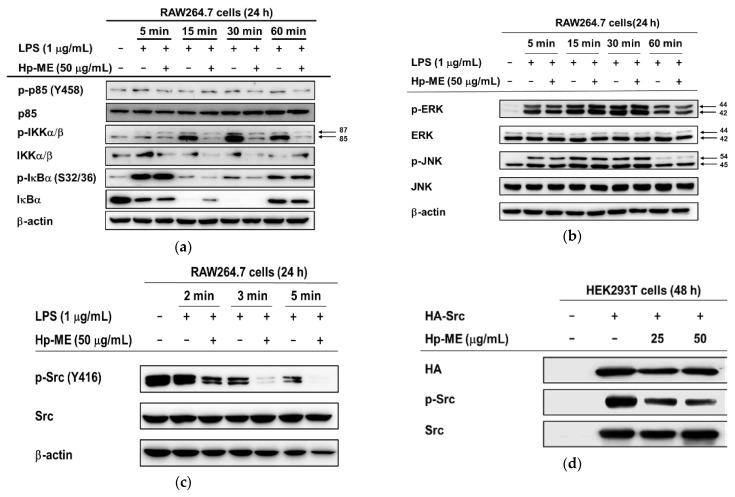

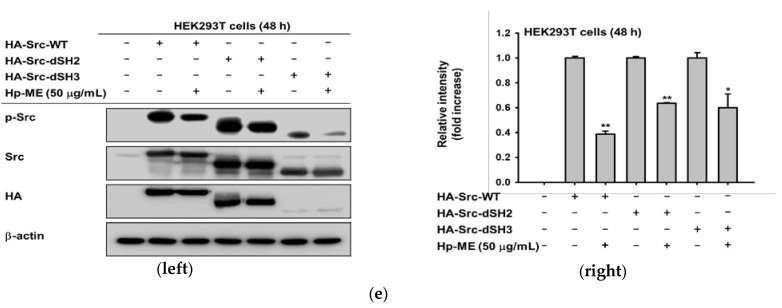
Efficacy of Hp-ME to inhibit activation of factors in the NF-κB pathway. (**a**) The phosphorylation levels of p85, IKKα/β, and IκBα and the expression levels of total proteins were confirmed in the whole lysate from macrophages treated with Hp-ME (50 μg/mL) and LPS (1 μg/mL) for 5 to 60 min. (**b**) The phosphorylation levels of ERK and JNK related to the AP-1 pathway and the expression levels of total proteins were assessed as for (**a**). (**c**) In the case of Src kinase, the LPS treatment was set from 2 to 5 min. (**d**) The phosphorylation degree and total protein level of Src kinase were determined in transfected HEK293T cells by HA-tagged plasmid. Twenty-four hours after gene transfection, Hp-ME treatment proceeded for a further 24 h. (**e**) After the transfection of each corresponding gene (HA-Src-WT, HA-Src-dSH2, or HA-Src-dSH3) and Hp-ME (50 μg/mL) treatment as in (**d**), the total protein level and the degree of phosphorylation in the whole lysate were confirmed (**left**). β-actin was used as a control protein. The fold increase was indicated by measuring the band intensity through the Image J program (**right**). For two groups transfected with the same gene, the band intensity of the Hp-ME-treated group was divided by the intensity of the untreated group (**right**). All data (**e**) (**right**) are presented as means ± standard deviation (SD) calculated from at least four independent samples. *: *p* < 0.05 and **: *p* < 0.01 compared to the Hp-ME untreated group.

To determine the target proteins of Hp-ME, first, we studied how the activity of factors in the NF-κB signaling pathway changes according to the presence or absence of Hp-ME. When Hp-ME was pretreated in RAW264.7 cells and then LPS was treated for 15 to 60 min, the phosphorylation of p85/PI3K and IKKα/β was greatly reduced. The phosphorylation level of IκBα, the downstream factor of IKKα/β, was significantly decreased at 15 and 30 min after LPS treatment (Figure 3a). To determine whether the target of Hp-ME is IKKα/β, we also checked the phosphorylation of Src, the most upstream signal of IKKα/β. In cells pretreated with Hp-ME and then treated with LPS for 2 to 5 min, the phosphorylation level of Src declined (Figure 3c). On the other hand, the phosphorylation of JNK and ERK, which are factors in the AP-1 pathway, and their total protein expression level, were not affected by Hp-ME (50 μg/mL) treatment (Figure 3b). Because of these outcomes, we checked the phosphorylation level in HEK293T cells overexpressing HA-Src and found that the activities of Src kinase declined at both 25 and 50 μg/mL (Figure 3d). Therefore, Hp-ME suppresses the phosphorylation of Src kinase to have an anti-inflammatory effect. To further investigate the domain of the Src kinase to which Hp-ME is attached, phosphorylation levels were determined when a specific domain was deleted. Since three domains (SH2 domain, SH3 domain, and kinase [SH1] domain) typically constitute Src kinase, HEK293T cells were transfected by the gene with the SH2 domain or SH3 domain ablated. However, with the specific domains ablated, a degree of phosphorylation was still abated (Figure 3e, left). When the SH2 domain was deleted, the degree of phosphorylation decreased to 60%, and when the SH3 domain was deleted, phosphorylation decreased to around 55% (Figure 3e, right). These results indicate that Hp-ME exhibits anti-inflammatory effects by binding to kinase domains rather than other domains.

### 2.4. Ulcerative Lesions Were Alleviated by Hp-ME in an HCl/EtOH-Induced Gastritis Mouse Model

**Figure 4:**
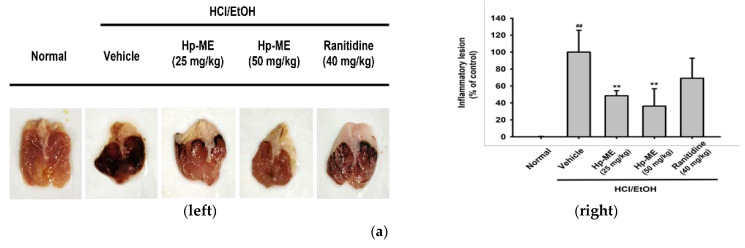

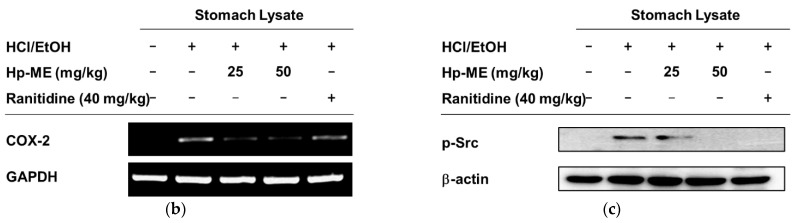
Hp-ME attenuated gastritis symptoms induced by HCl/EtOH. (**a**) Mice were given oral injections of Hp-ME (0–50 mg/kg) or ranitidine (40 mg/kg) three times at intervals of 8 h. Gastritis was induced with HCl/EtOH (150 mM) 1 h before mice were sacrificed. The stomach was removed from the sacrificed mice and cut so that the inner surface was visible. Photographs of damaged lesions were taken for each group using a digital camera (**left**). The Image J program was used to quantify and display the degree of damage and alleviation due to gastritis (**right**). (**b**) The band intensity indicated mRNA expression level of COX-2 in stomach tissue lysate. GAPDH was checked as a control gene. (**c**) The phosphorylation level of Src kinase was also confirmed from stomach tissue lysate. β-actin was checked as a control protein. All data (**a**) (right) are presented as means ± standard deviation (SD) calculated from at least five mice. ##: *p* < 0.01 compared to the non-induced group, and **: *p* < 0.01 compared to the control group (gastritis induced by HCl/EtOH).

To confirm the anti-inflammatory effect of Hp-ME in vivo, gastritis was induced in mice by oral administration of HCl/EtOH. The ulcerative lesions were alleviated by the administration of Hp-ME (0–50 mg/kg) (Figure 4a, left). Hp-ME was excellent in alleviating inflammation when compared to ranitidine, which is the standard anti-ulcer drug. When the ulcerative lesions were measured numerically, they were reduced to about 50% at the 25 mg/kg of Hp-ME and to about 37% at 50 mg/kg (Figure 4a, right). In addition, as we have confirmed in vitro, not only mRNA expression level of COX-2 but also the phosphorylation of Src was suppressed by Hp-ME (Figure 4b,c). This result shows that Hp-ME can inhibit inflammation in HCl/EtOH-induced gastritis.

### 2.5. Lung Injury Was Attenuated by Hp-ME in an LPS-Induced ALI Mouse Model

**Figure 5:**
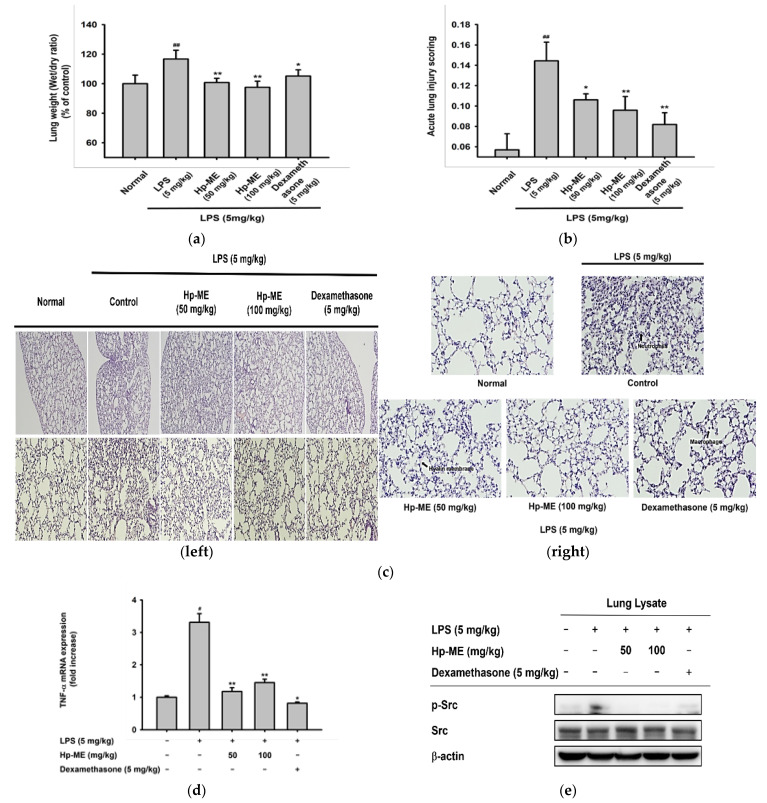
Hp-ME inhibited ALI induced by LPS. (**a**) Mice were given an oral instillation of Hp-ME (0–100 mg/kg) or dexamethasone (5 mg/kg) two times before LPS induction and once after induction. After 16 h of induction, the wet-to-dry ratios for each group were averaged. (**b**) According to the Lung Injury Scoring System of the American Thoracic Society, the degree of acute lung injury was scored using H&E staining data of the upper right lobe. (**c**) Under the microscope [4× and 10× magnification (**left**), or 20× magnification (**right**)], neutrophils, formation of hyaline membrane, type II pneumocyte hyperplasia, and thickening of alveolar septa were observed. (**d**) The mRNA expression level of TNF-α was determined in the lysate of the lower right lobe by quantitative real-time PCR. GAPDH was checked as a control gene. (**e**) The phosphorylation level of Src kinase was also demonstrated from lung tissue lysate. β-actin was checked as a control protein. All data (**a**,**b**,**d**) are presented as means ± standard deviation (SD) calculated from at least five mice or samples. #: *p* < 0.05 and ##: *p* < 0.01 compared to the non-induced group, and *: *p* < 0.05 and **: *p* < 0.01 compared to the control group (ALI induced by LPS).

Pulmonary edema is the most important pathological feature to consider in ALI. Therefore, first, we measured and calculated the wet-to-dry weight in the left lobe of each mouse. Compared with the normal group, the wet-to-dry weight increased about 1.2 times in the group with ALI induced by LPS. On the other hand, the weight ratio of the Hp-ME-treated group decreased to the same level as the normal group (Figure 5a). The degree of pulmonary edema was further reduced compared to the group treated with dexamethasone. Dexamethasone is known to alleviate ALI by reducing the filtration of LPS-induced neutrophils as well as the production of TNF-α [14]. These results indicate that Hp-ME can alleviate LPS-induced pulmonary edema in mice. Second, histological data of the upper right lobe was confirmed as the referential feature of ALI (Figure 5c). The histological evidence of tissue injury was measured by referring to the Lung Injury Scoring System of the American Thoracic Society [15]. Scores were designated by the number of neutrophils in the alveolar space or interstitial space, hyaline membranes, and alveolar septal thickening in each field. A total of four fields were determined per tissue and the indicators were checked. Based on these results, the scores were calculated for each tissue according to the equation described in the methods (Table 1). When the scores were averaged for each group, the control group with acute lung damage caused by LPS (5 mg/kg) had an average of about 2.5 times higher than that of the normal group. The average score decreased to around 66% depending on the concentration of Hp-ME, and it was almost the same as that of the group fed dexamethasone (5 mg/kg) orally (Figure 5b). This indicates that Hp-ME can prevent the increase of neutrophil infiltration, hyaline membrane, and thickening of alveolar septa. In addition, it was confirmed by H&E staining that alveolar septal fibrosis and type II pneumocyte hyperplasia in mice treated with Hp-ME were weaker than in the group treated with LPS alone. The phenomena so far are characteristic of diffused alveolar damage, which is a representative histological hallmark of ALI [16]. Finally, as in the results confirmed in vitro, not only the mRNA level of TNF-α but also the phosphorylation of Src, the target protein of Hp-ME was decreased in the group fed Hp-ME (50–100 mg/kg) orally (Figure 5d,e). Therefore, these results show that Hp-ME has anti-inflammatory effects in an LPS-induced ALI mouse model.

## 3. Discussion

When pathogen infection and tissue damage are generated, inflammation occurs as a physiological defense mechanism. This system stops as soon as circumstances return to normal [17]. Thus, the inflammatory response includes the sequential release of mediators and the recruitment of activated immune cells. After that, anti-inflammatory cytokines and intracellular negative regulatory factors are used at an appropriate time to clear the inflammatory response after recovery [18]. If repairs continue without phasing properly even when a healthy state is resumed, permanent tissue damage can be caused by the unregulated production of leukocytes, lymphocytes, and related cytokines [19,20]. Therefore, studies have focused on the mechanisms that regulate the inflammatory response and the materials with the potential to control it.

Hp-ME is the highest-ranked among the herbal extracts in our laboratory library that can inhibit the effects of NO production. NO is a mediator that is generated in inflammatory reactions and has been studied as a target in relation to drug development [21]. Based on its NO production and inhibitory effect, the possibility of Hp-ME as a drug capable of controlling unbalanced inflammatory responses was confirmed in this paper.

**Figure 6:**
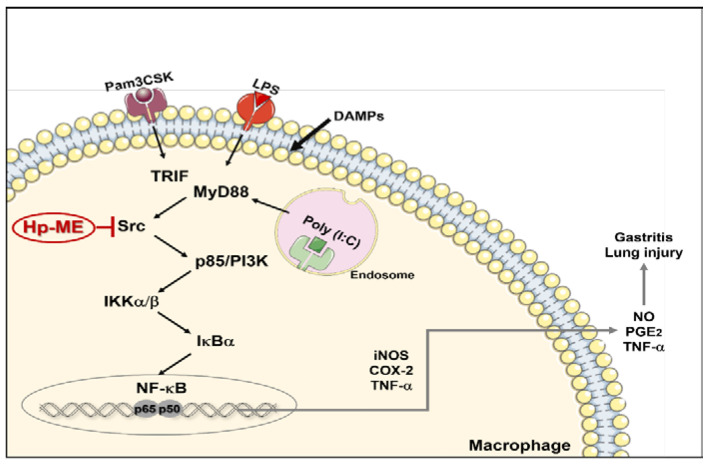
A scheme of the overall mechanism by which Hp-ME could exhibit anti-inflammatory efficacy.

The NO reducing effect of Hp-ME was examined in macrophages stimulated with Pam3CSK (ligand of TLR1/2) or poly(I:C) (ligand of TLR3) as well as LPS (ligand of TLR4). This effect was confirmed by comparing Aa-EE with Hp-ME, an oral drug for the treatment of gastric mucosal lesions of acute gastritis [22]. In addition, through LC-MS/MS analysis, Hp-ME was revealed to have several flavonoids (5-methyl kaempferol, genistein1, and penduletin) that are reported to ameliorate inflammation [23,24,25]. The diminution in the final product of inflammation leads to repression in the mRNA expression of related genes as well. After Hp-ME treatment, the mRNA levels of COX-2 producing inflammatory factor PGE2, iNOS producing NO, and inflammatory factor TNF-α were suppressed [26,27]. In this regard, a luciferase assay was used to verify that mRNA levels were decreased due to the regulation of transcription factors. When MyD88 and TRIF (adaptor molecules of TLRs) are overexpressed, Hp-ME suppressed the activities of transcription factors. In addition, the protein expression levels of the subunits of nuclear factor (NF)-κB factor, which is responsible for regulating the transcription of inflammation-related genes, were also suppressed [28]. Therefore, the phosphorylation of Src kinase, p85/PI3K, IKKα/β, and IκBα included in the NF-κB pathway was inhibited in macrophages treated with Hp-ME. Based on the results so far, it could be revealed that Hp-ME suppresses the inflammatory signal by targeting Src kinase, which is the initial signaling factor. Src kinase is a proto-oncogene, which plays an important role in regulating not only cellular responses but also inflammatory responses (Figure 6) [29]. In particular, Hp-ME interferes with the phosphorylation site containing Tyr residue 416 in the protein kinase domain of Src and explains how Hp-ME could have the ability to alleviate acute gastritis induced by HCl/EtOH. ALI induced by LPS was also relieved by Hp-ME along with lung edema.

Both in vitro and in vivo experiments revealed the potential of Hp-ME as a therapeutic agent for acute inflammation. In the animal model, more detailed studies are needed on the immune activities that can result from the action of Hp-ME, this would further enhance its potential. Finally, during the screening, Hp-ME has radical scavenging ability in DPPH and ABTS assays, which is frequently used to evaluate antioxidant capacities in plant extracts [30]. Therefore, if further research on the antioxidant function of Hp-ME is conducted, it can be used as a competent therapeutic component in various fields.

## 4. Materials and Methods

### 4.1. Materials and Reagents

Hp-ME was supplied from the International Biological Material Extract Bank (Daejeon, Korea). RAW264.7 cells (ATCC number TIB-71) and HEK293T cells (ATCC number CRL-1573) were purchased from the American Type Culture Collection (ATCC) (Rockville, MD, USA). A 95% ethanol extract (Code no.: CA02-070) of *Artemisia* *asiatica* was purchased from the Plant Extract Bank of the Plant Diversity Research Center (http://extract.pdrc.re.kr/extract/f.htm, 25 October 2021), Daejeon, Korea, as reported previously [22]. Roswell Park Memorial Institute (RPMI) 1640 medium, Dulbecco’s modified Eagle’s medium (DMEM), fetal bovine serum (FBS), penicillin, streptomycin, phosphate-buffered saline (PBS), and TRIzol reagent were purchased from GIBCO (Grand Island, NY, USA). LPS, (3–4,5-dimethylthiazol-2-yl)-2,5-diphenyl-tetrazolium bromide (MTT), ranitidine, Nω-Nitro-L-arginine methyl ester hydrochloride (L-NAME), Pam3CSK4 (Pam3), poly(I:C), polyethylenimine (PEI), sodium dodecyl sulfate (SDS), and dexamethasone were purchased from Sigma Chemical Co. (St. Louis, MO, USA). Total or phospho-specific antibodies against p50, p65, IκBα, IKKα/β, Src, LaminA/C, JNK, ERK, and β-actin were obtained from Cell Signaling Technology (Beverly, MA, USA). Enhanced chemiluminescence (ECL) reagents were purchased from Ab Frontier (Seoul, Korea).

### 4.2. Plant Extraction

*Huberia peruviana* Cogn. was collected in San Juan de Punchis district, Zamora Chinchipe province in Ecuador and identified by Bolivar Merino, herbarium of Loja National University in 2012. A voucher specimen (accession number KRIB 0044369) of the retained material is preserved at the herbarium of KRIBB. The leaves of *Huberia peruviana* Cogn. (30 g) was extracted with 1 L of 99.9% (*v*/*v*) methanol repeating sonication (15 min) and resting (2 h) for three days at 45 °C as reported previously [31]. The resultant product was filtered with non-fluorescence cottons, and concentrated by rotary evaporator (N-1000SWD, EYELA) under reduced pressure at 45 °C. Finally, total 4.56 g of methanol extract of *Huberia peruviana* Cogn. was obtained by freeze-drying.

### 4.3. Cell Culture

Murine macrophage RAW264.7 cells were cultured at a density of 80–90% in 10-cm cell culture dishes using RPMI 1640 medium containing 1% penicillin, streptomycin, and 10% FBS at 37 °C in 5% CO_2_. Human cell line HEK293T cells were also cultured at a density of 70–80% in a 10-cm cell culture dish using DMEM medium containing 1% penicillin/streptomycin and 10% FBS at 37 °C and 5% CO_2_.

### 4.4. Nitric Oxide (NO) Production Assay

RAW264.7 cells were adjusted to a concentration of 1 × 10^6^ cell/mL using RPMI 1640 medium, and then inoculated (100 μL per well) into 96-well plates and cultured for 18 h at 37 °C in 5% CO_2_. The cells were treated with 50 μL of the Hp-ME, Aa-EE, a standard herbal medicine [32], or L-NAME, an inhibitor of NOS, prepared at four-fold concentrations. After 30 min, cells were treated with 50 μL of LPS (target concentration 1 μg/mL) or Pam3CSK4 or Poly(I:C). After 24 h, the absorbance was measured at 540 nm after mixing 1 to 1 with Griess solution for 100 μL of the supernatant.

### 4.5. Liquid Chromatography–Mass Spectrometry (LC-MS)

To perform the LC-MS/MS analysis, Xevo G2-XS Q-TOF-LC/MS (Waters, Milford, MA, USA) was used as reported previously [33]. A reverse-phase BEH C18, 2.1 × 100 mm, 1.7 μm column (Waters, Milford, MA, USA) was used for UPLC. The mobile phases were (A) 0.1% formic acid in water and (B) acetonitrile. The UPLC conditions were as follows: 0 min 80:20 linear gradient to 5:95 in 14 min, 14 min 5:95 linear gradient to 0:100 in 15 min, 15 min 0:100 linear gradient to 80:20 in 15.1 min and held for 2.9 min. The detailed conditions were set up as described previously [32]. From Waters LC-MS-QTOF MassLynx Software version 4.2 and Waters UNIFI Portal Software (Waters, Milford, MA, USA), data were acquired and analysis was performed, respectively.

### 4.6. Cell Viability Assay

RAW264.7 cells were cultured for 18 h at 37 °C in 5% CO_2_. The cells were treated with 50 μL of the Hp-ME or standard compound (Aa-EE, L-NAME). After 24 h, 10 μL MTT solution (stock concentration: 5 mg/mL) was added and a further reaction was induced for 3 h. MTT stopping solution (100 μL, 10% SDS in 0.01 M HCl) was also added to each well to terminate the reaction and dissolve the formazan crystal. After 24 h, the absorbance was measured at 540 nm.

### 4.7. Analysis of mRNA Expression Level Using Semiquantitative and Quantitative Reverse Transcription-Polymerase Chain Reaction (RT-PCR)

RAW264.7 cells were seeded in a 12-well plate and pretreated with Hp-ME for 30 min. After treating the LPS for 6 h, the media were discarded. Total RNA was extracted from the cells with TRIzol reagent and then cDNA was synthesized from 1 μg of total RNA using MuLV RT according to the manufacturer’s instructions (Thermo Fisher Scientific, Waltham, MA, USA). The total RNA and synthesized cDNA were also obtained from stomach tissues of HCl/EtOH-induced gastritis mice or lung tissues of ALI mice. Synthesized cDNA is used for semiquantitative and quantitative RT-PCR as reported previously [34,35]. The primer sequences are listed in Table 2 and Table 3.

### 4.8. Luciferase Reporter Gene Activity Assay

After adjusting HEK293T cells to a concentration of 2 × 10^5^ cell/mL using DMEM medium, they were plated on 24-well plates and incubated at 37 °C in 5% CO_2_ for 18 h. The cells were co-transfected with adaptor molecule genes (MyD88 or TRIF) with the NF-κB–Luc (luciferase) gene and β-galactosidase using PEI as reported previously [36]. After 24 h, cells were treated with Hp-ME (0–50 μg/mL). After an additional 24 h, the luciferase activity was detected using luciferin by luminescence and normalized to β-galactosidase activity using a luminometer.

### 4.9. Preparation of Total Cell and Nuclear Lysates

RAW264.7 cells were cultured on 3-cm plates for 18 h. Hp-ME and LPS were added in order by the method mentioned previously. Cells were collected in cold PBS and broken with a cell lysis buffer. After centrifuging at 12,000 rpm for 1 min, the supernatant was used. In the case of nuclear lysates, the cells were broken with a homogenization buffer A (20 mM Tris-HCl (pH 8.0), 10 mM EGTA, 2 mM EDTA, 2 mM DTT, 1 mM PMSF, 25 μg/mL aprotinin, 10 μg/mL leupeptin). After sonicating and centrifuging, the pellet was resolved in homogenization buffer B (buffer A, 1% Triton X-100). In the case of HEK293T cells, after transfection with specific genes for 24 h, the cells were broken with a cell lysis buffer.

### 4.10. Western Blotting Analysis

Protein samples in total cell lysates, nuclear lysates, stomach lysate for gastritis model, and lung lysate for ALI model were separated by SDS-polyacrylamide gel electrophoresis (Bio-Rad, Hercules, CA, USA), and western blotting analysis was executed with specific antibodies for each target protein, as reported previously [37].

### 4.11. In Vivo HCl/EtOH-Induced Acute Gastritis Mouse Model

ICR mice (*n* = 5/group) were injected by HCl/EtOH to induce an acute gastritis mouse model. Fasted ICR mice were treated orally with Hp-ME (25 and 50 mg/kg) or ranitidine (40 mg/kg) three times every 12 h. At 8 h after the final administration, mice received 300 μL of 60% ethanol in 150 mM HCl orally to induce acute gastritis. After 1 h, mice were anesthetized with isoflurane and sacrificed. The stomachs were extracted and washed in PBS, with images obtained of the inner lesions. This gastritis in vivo study was conducted according to the guidelines of the Institutional Animal Care and Use Committee at Sungkyunkwan University (Suwon, Korea; approval ID: SKKUIACUC2020-03-29-1).

### 4.12. In Vivo LPS-Induced ALI Model

C57BL/6 mice (*n* = 5/group) received LPS intranasally to induce the ALI model. Hp-ME (50 and 100 mg/kg) and dexamethasone (5 mg/kg) were administered twice orally 7 h and 1 h before LPS induction. After that, intranasal injection of LPS (5 mg/kg) in 50 μL volumes were administered twice every 30 min. After 1 h, the last oral instillation of Hp-ME or dexamethasone was performed. After fasting for 16 h mice were anesthetized with isoflurane and sacrificed. The extracted lungs were divided into three parts. The left side of the lungs was used to measure wet/dry lung weight. Thus, the wet left side was weighed and dried at 75 °C in a drying oven for 48 h. To calculate the wet/dry lung weight ratio (W/D) which is mainly used to reflect the degree of pulmonary edema, dried lung tissue was also weighed. The upper right side was used to study histological change. Thus, this tissue was fixed in 4% paraformaldehyde for 24 h and then embedded in paraffin. The lungs were cut into sections 4 μm thick. To check pathological change using the number of neutrophils and hyaline membranes, the sections were stained with hematoxylin-eosin. The lower right side of the lung was used in a western blotting assay or mRNA expression level analysis. This ALI in vivo study was conducted according to the guidelines of the Institutional Animal Care and Use Committee at Sungkyunkwan University (Suwon, Korea; approval ID: SKKUIACUC2020-12-44-1).

### 4.13. Statistical Analysis

All data used in this study are presented as means ± standard deviation (SD) calculated from at least four independent samples. To analyze the statistical significance of the difference between values for the various experimental groups and control group, a Student’s t-test and Mann–Whitney test were used and a *p*-value < 0.05 was considered statistically significant.

## 5. Conclusions

In summary, this study suggested that Hp-ME has an anti-inflammatory effect by targeting Src kinase in macrophages activated by pathogens. This inhibits mRNA levels of inflammation-related cytokines, enzymes, the activity of transcription factors, and the signaling system of the NF-κB pathway. In an animal model, Hp-ME also exhibited the ability to alleviate inflammation in acute gastritis induced by HCl/EtOH and ALI induced by LPS. These results demonstrated that Hp-ME could be exploited as an anti-inflammatory drug in many inflammatory diseases.

## Figures and Tables

**Table 1:** Lung injury scoring parameter.

Parameter	Score per Field		
	0	1	2
A. Neutrophils in the alveolar space	None	1–5	>5
B. Neutrophils in the interstitial space	None	1–5	>5
C. Hyaline membranes	None	3	>3
D. Alveolar septal thickening	<2X	2X–4X	>4X
Score=[(20×A)+(14×B)+(7×C)+(2×D)]/(numberoffields×100)			

**Table 2:** Sequences of primers used in semiquantitative RT-PCR analysis.

Gene	Direction	Sequences (5′ → 3′)
iNOS	Forward Reverse	TGCCAGGGTCACAACTTTACA ACCCCAAGCAAGACTTGGAC
COX-2	Forward Reverse	TGAGTACCGCAACGCTTCT TGGGAGGCACTTGCATTGAT
TNF-α	Forward Reverse	TTGACCTCAGCGCTGAGTTG CCTGTAGCCCACGTCGTAGC
GAPDH	Forward Reverse	GAAGGTCGGTGTGAACGGAT AGTGATGGCATGGACTGTGG

**Table 3:** Sequences of primers used in quantitative RT-PCR analysis.

Gene	Direction	Sequences (5′ → 3′)
TNF-α	Forward Reverse	TGCCTATGTCTCAGCCTCTT GAGGCCATTTGGGAACTTCT
GAPDH	Forward Reverse	TGTGAACGGATTTGGCCGTA ACTGTGCCGTTGAATTGCC

## Data Availability

The data used to support the findings of this study are available from the corresponding author upon request.
